# Supplementary material for: A yeast phenomic model for the influence of Warburg metabolism on genetic buffering of doxorubicin
Source: Cancer Metab. 2019 Oct 23;7:9. doi: 10.1186/s40170-019-0201-3 (PMC6806529; doi:10.1186/s40170-019-0201-3)
Supplement: Supplementary file 9 — Additional file 9. GO term-specific heatmaps for REMc/GTF-enriched clusters. GO term-specific heatmaps for significant GO process terms were generated as described in methods and Figs. 3 and 4. Any related child terms are presented in subsequent pages of the parent file name. GO terms with more than 100 children, with 2 or fewer genes annotated to the term, or a file size over 300KB are not shown. All heatmaps are generated with the same layout (see Figs. 3 and 4). [file 40170_2019_201_MOESM9_ESM.bz2 › Additional_File9_GOTermHeatmaps/Additional_File9_GOTermHeatmap/RNA_3'-end_processing.pdf]

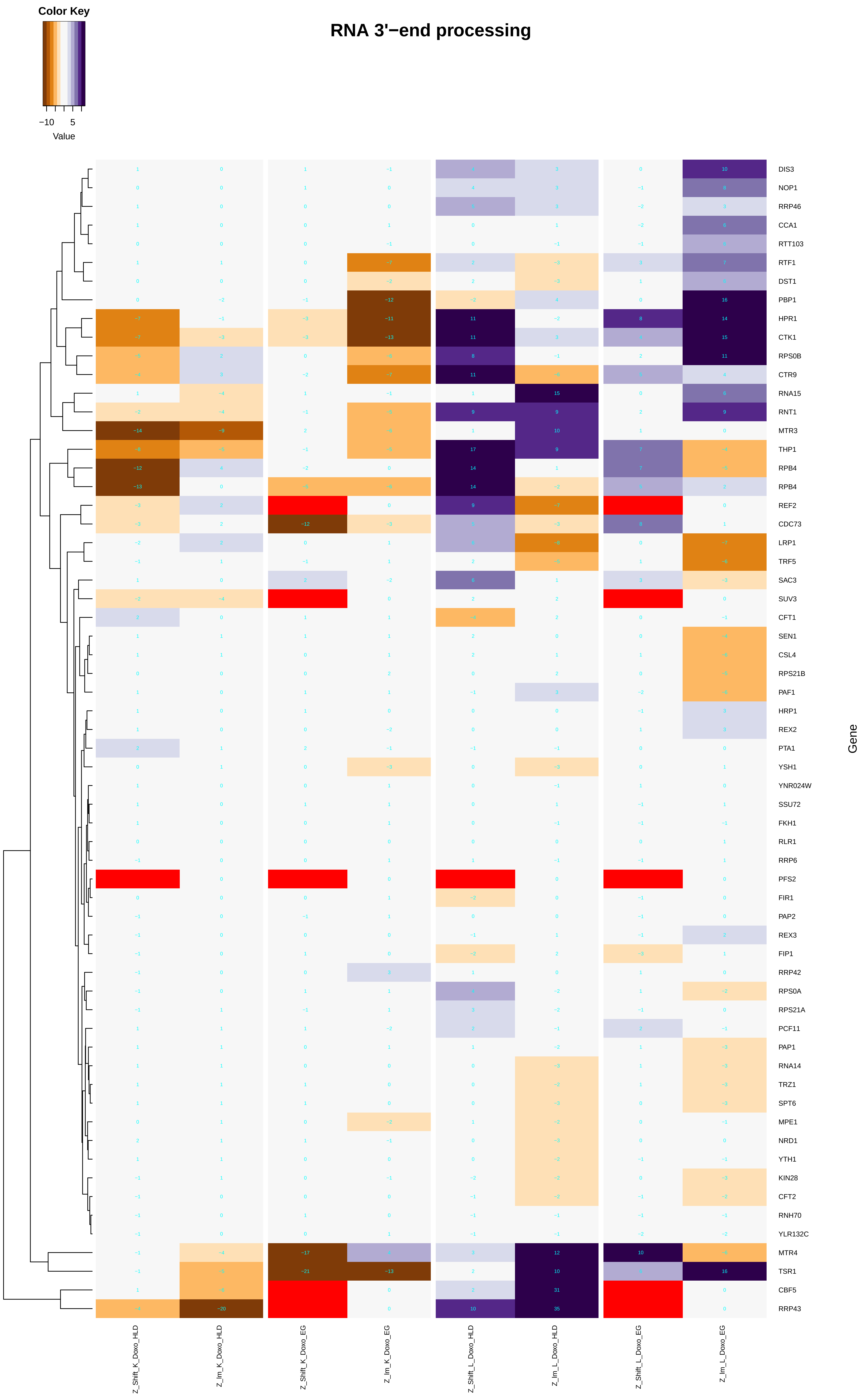
Color Key
−10
5
Value
RNA 3'−end processing
DIS3
NOP1
RRP46
CCA1
RTT103
RTF1
DST1
PBP1
HPR1
CTK1
RPS0B
CTR9
RNA15
RNT1
MTR3
THP1
RPB4
RPB4
REF2
CDC73
LRP1
TRF5
SAC3
SUV3
CFT1
SEN1
CSL4
RPS21B
PAF1
HRP1
REX2
PTA1
YSH1
YNR024W
SSU72
FKH1
RLR1
RRP6
PFS2
FIR1
PAP2
REX3
FIP1
RRP42
RPS0A
RPS21A
PCF11
PAP1
RNA14
TRZ1
SPT6
MPE1
NRD1
YTH1
KIN28
CFT2
RNH70
YLR132C
MTR4
TSR1
CBF5
RRP43
Gene
Z_Shift_K_Doxo_HLD
Z_Im_K_Doxo_HLD
Z_Shift_K_Doxo_EG
Z_Im_K_Doxo_EG
Z_Shift_L_Doxo_HLD
Z_Im_L_Doxo_HLD
Z_Shift_L_Doxo_EG
Z_Im_L_Doxo_EG

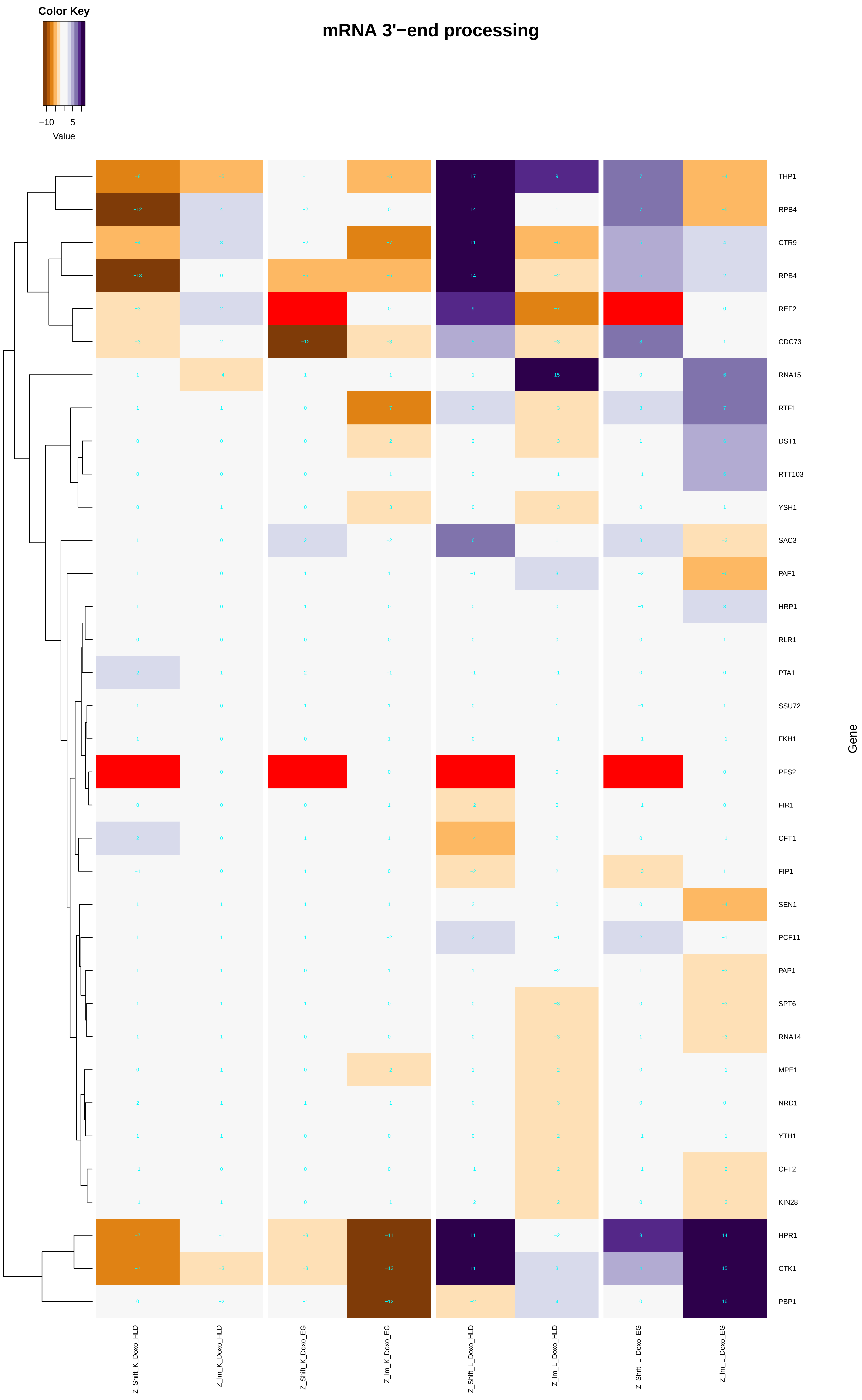

mRNA 3'−end processing
Color Key
−10
5
Value
THP1
RPB4
CTR9
RPB4
REF2
CDC73
RNA15
RTF1
DST1
RTT103
YSH1
SAC3
PAF1
HRP1
RLR1
PTA1
SSU72
FKH1
PFS2
FIR1
CFT1
FIP1
SEN1
PCF11
PAP1
SPT6
RNA14
MPE1
NRD1
YTH1
CFT2
KIN28
HPR1
CTK1
PBP1
Gene
Z_Shift_K_Doxo_HLD
Z_Im_K_Doxo_HLD
Z_Shift_K_Doxo_EG
Z_Im_K_Doxo_EG
Z_Shift_L_Doxo_HLD
Z_Im_L_Doxo_HLD
Z_Shift_L_Doxo_EG
Z_Im_L_Doxo_EG

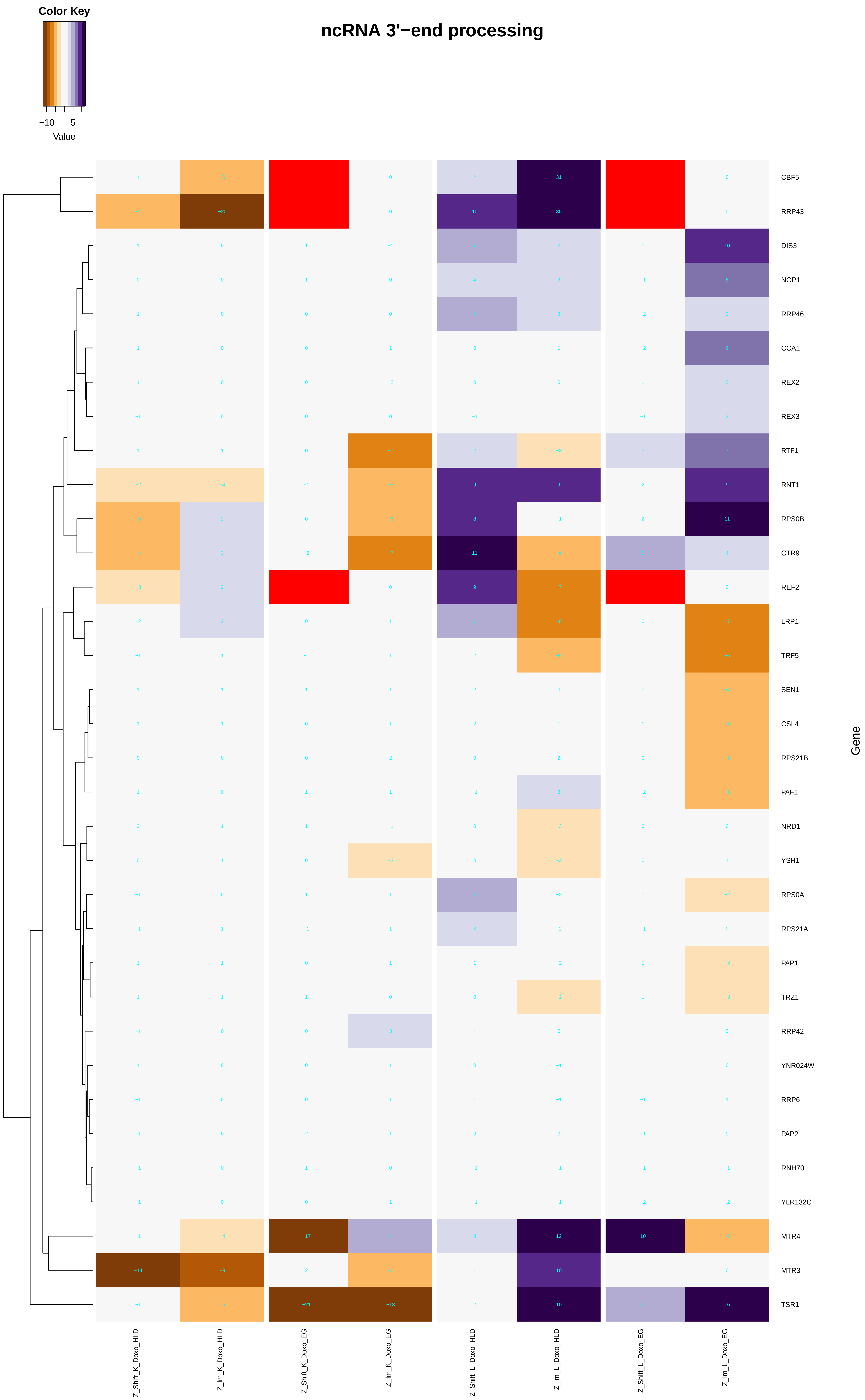

Color Key
−10
5
Value
ncRNA 3'−end processing
CBF5
RRP43
DIS3
NOP1
RRP46
CCA1
REX2
REX3
RTF1
RNT1
RPS0B
CTR9
REF2
LRP1
TRF5
SEN1
CSL4
RPS21B
PAF1
NRD1
YSH1
RPS0A
RPS21A
PAP1
TRZ1
RRP42
YNR024W
RRP6
PAP2
RNH70
YLR132C
MTR4
MTR3
TSR1
Gene
Z_Shift_K_Doxo_HLD
Z_Im_K_Doxo_HLD
Z_Shift_K_Doxo_EG
Z_Im_K_Doxo_EG
Z_Shift_L_Doxo_HLD
Z_Im_L_Doxo_HLD
Z_Shift_L_Doxo_EG
Z_Im_L_Doxo_EG

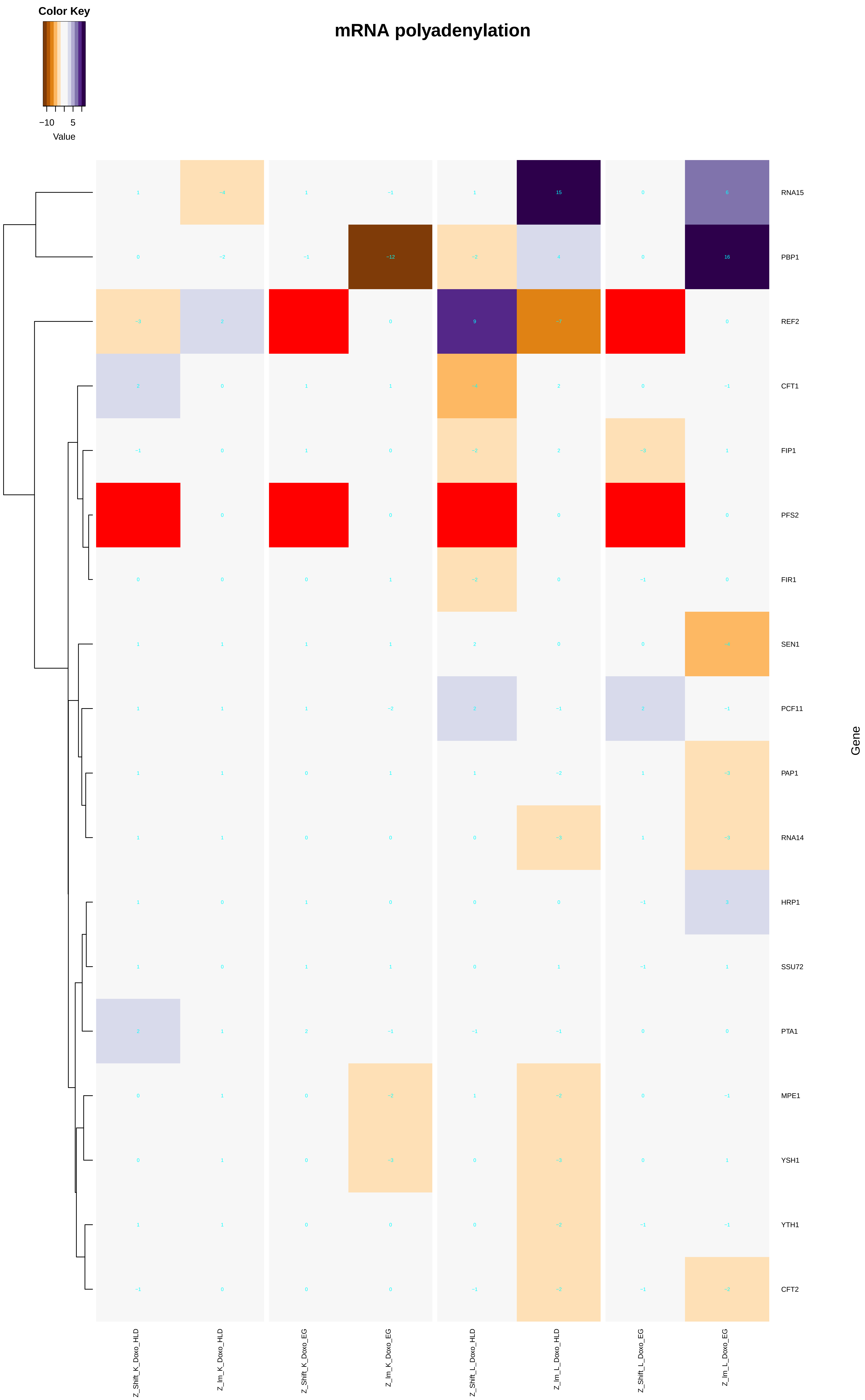
Color Key
−10
5
Value
mRNA polyadenylation
RNA15
PBP1
REF2
CFT1
FIP1
PFS2
FIR1
SEN1
PCF11
PAP1
RNA14
HRP1
SSU72
PTA1
MPE1
YSH1
YTH1
CFT2
Gene
Z_Shift_K_Doxo_HLD
Z_Im_K_Doxo_HLD
Z_Shift_K_Doxo_EG
Z_Im_K_Doxo_EG
Z_Shift_L_Doxo_HLD
Z_Im_L_Doxo_HLD
Z_Shift_L_Doxo_EG
Z_Im_L_Doxo_EG

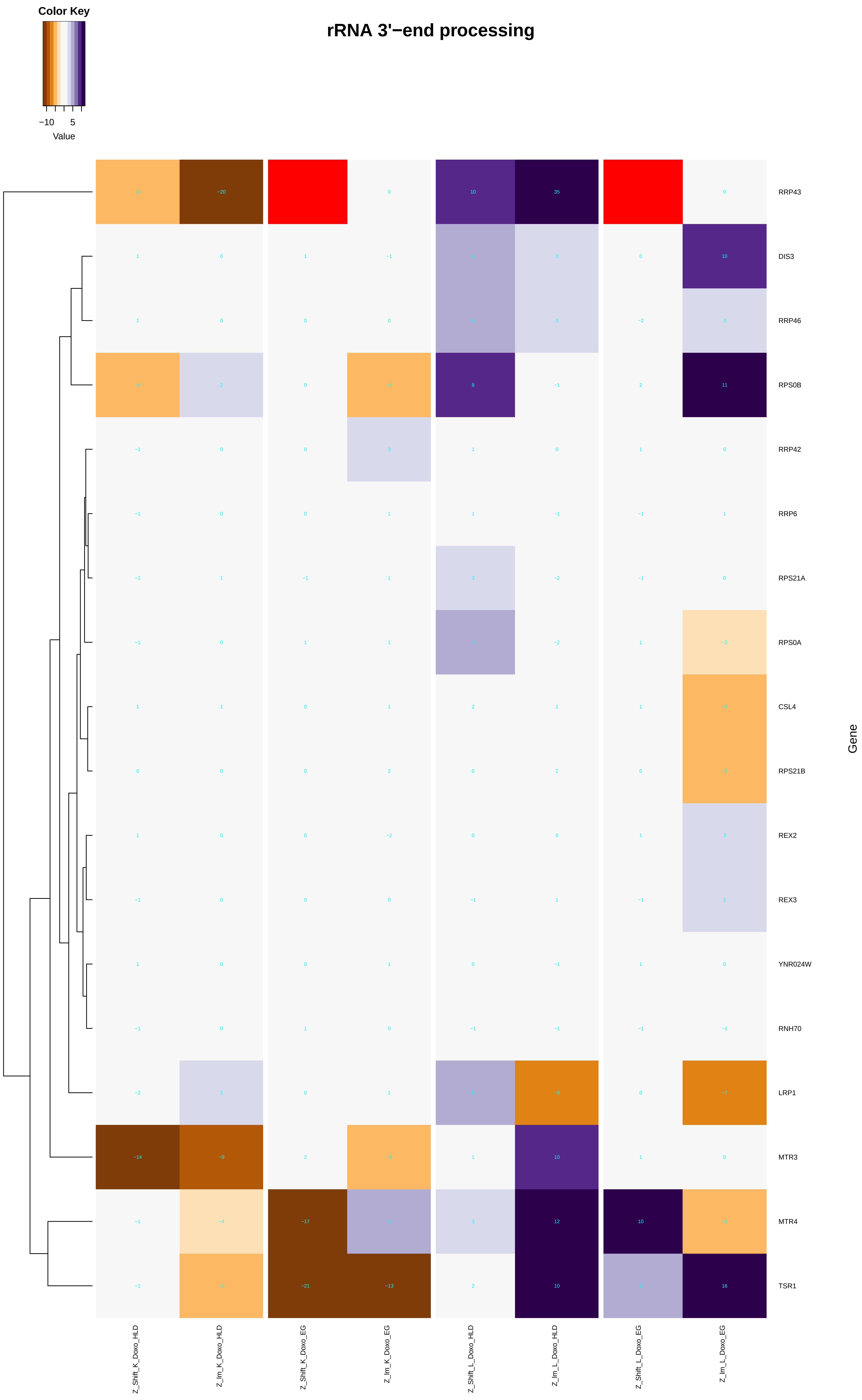
rRNA 3'−end processing
Color Key
−10 5
Value
RRP43
DIS3
RRP46
RPS0B
RRP42
RRP6
RPS21A
RPS0A
CSL4
RPS21B
REX2
REX3
YNR024W
RNH70
LRP1
MTR3
MTR4
TSR1
Gene
Z_Shift_K_Doxo_HLD
Z_Im_K_Doxo_HLD
Z_Shift_K_Doxo_EG
Z_Im_K_Doxo_EG
Z_Shift_L_Doxo_HLD
Z_Im_L_Doxo_HLD
Z_Shift_L_Doxo_EG
Z_Im_L_Doxo_EG

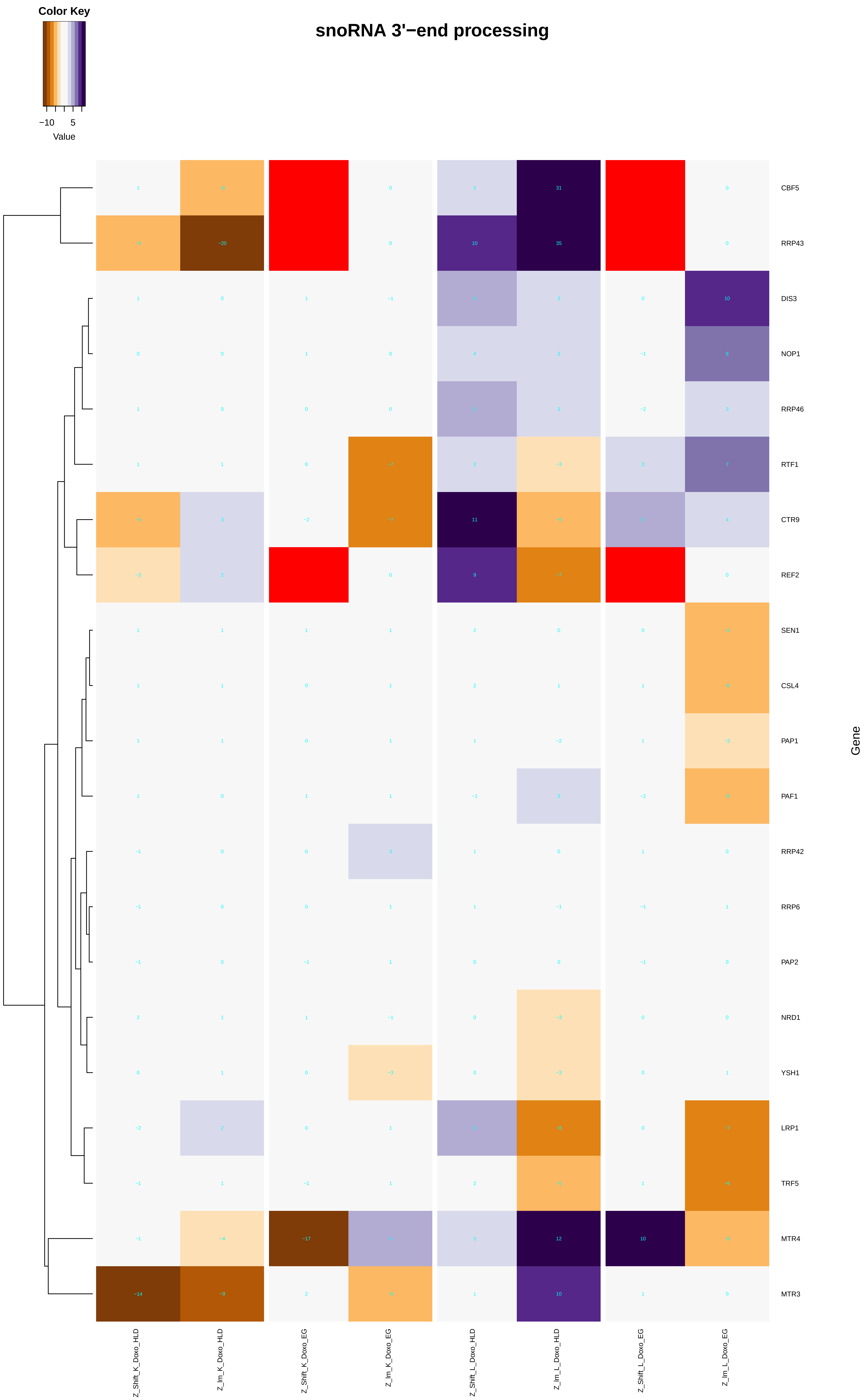
snoRNA 3'−end processing
Color Key
−10
5
Value
CBF5
RRP43
DIS3
NOP1
RRP46
RTF1
CTR9
REF2
SEN1
CSL4
PAP1
PAF1
RRP42
RRP6
PAP2
NRD1
YSH1
LRP1
TRF5
MTR4
MTR3
Gene
Z_Shift_K_Doxo_HLD
Z_Im_K_Doxo_HLD
Z_Shift_K_Doxo_EG
Z_Im_K_Doxo_EG
Z_Shift_L_Doxo_HLD
Z_Im_L_Doxo_HLD
Z_Shift_L_Doxo_EG
Z_Im_L_Doxo_EG

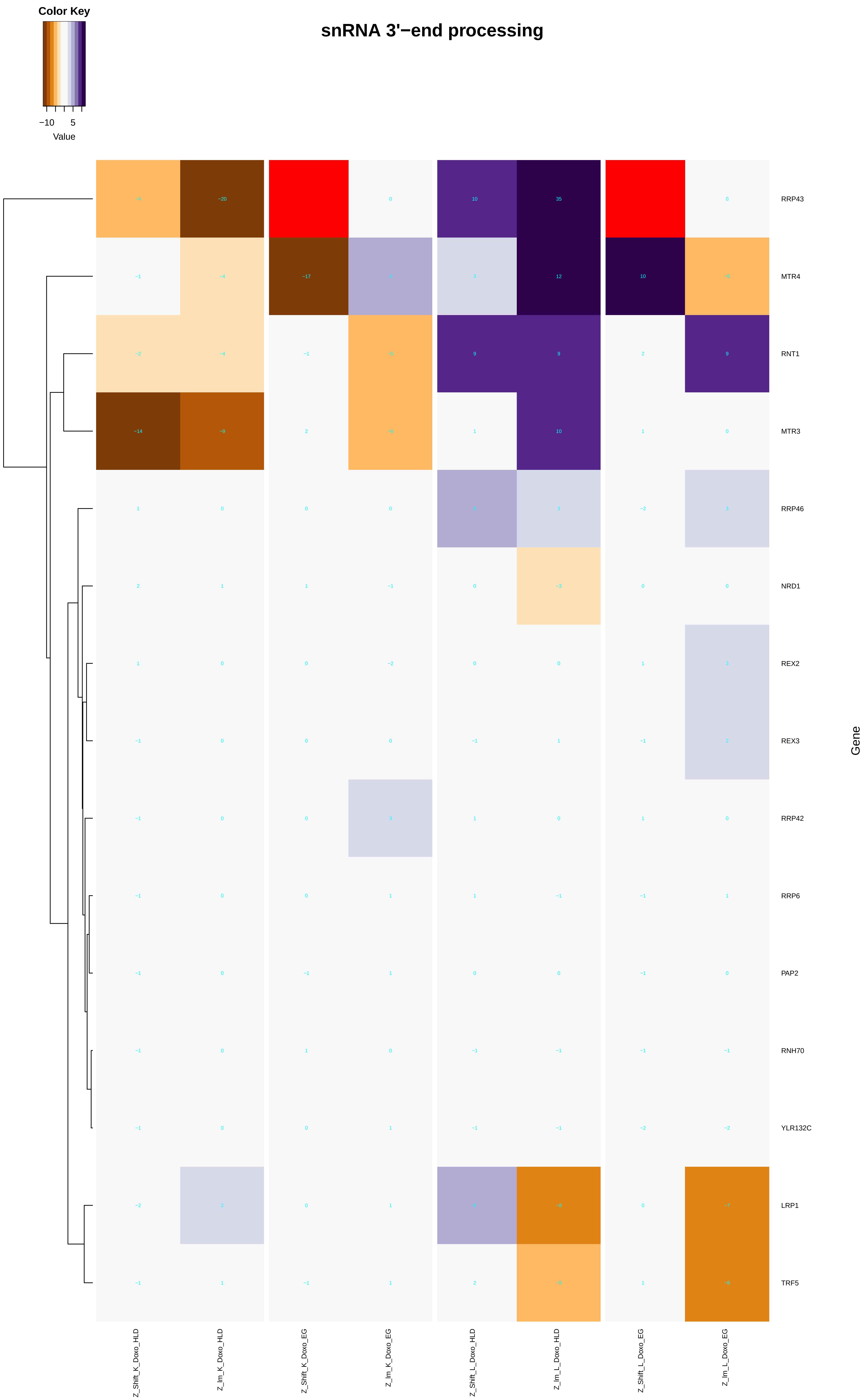

Color Key
−10
5
Value
snRNA 3'−end processing
RRP43
MTR4
RNT1
MTR3
RRP46
NRD1
REX2
REX3
RRP42
RRP6
PAP2
RNH70
YLR132C
LRP1
TRF5
Gene
Z_Shift_K_Doxo_HLD
Z_Im_K_Doxo_HLD
Z_Shift_K_Doxo_EG
Z_Im_K_Doxo_EG
Z_Shift_L_Doxo_HLD
Z_Im_L_Doxo_HLD
Z_Shift_L_Doxo_EG
Z_Im_L_Doxo_EG

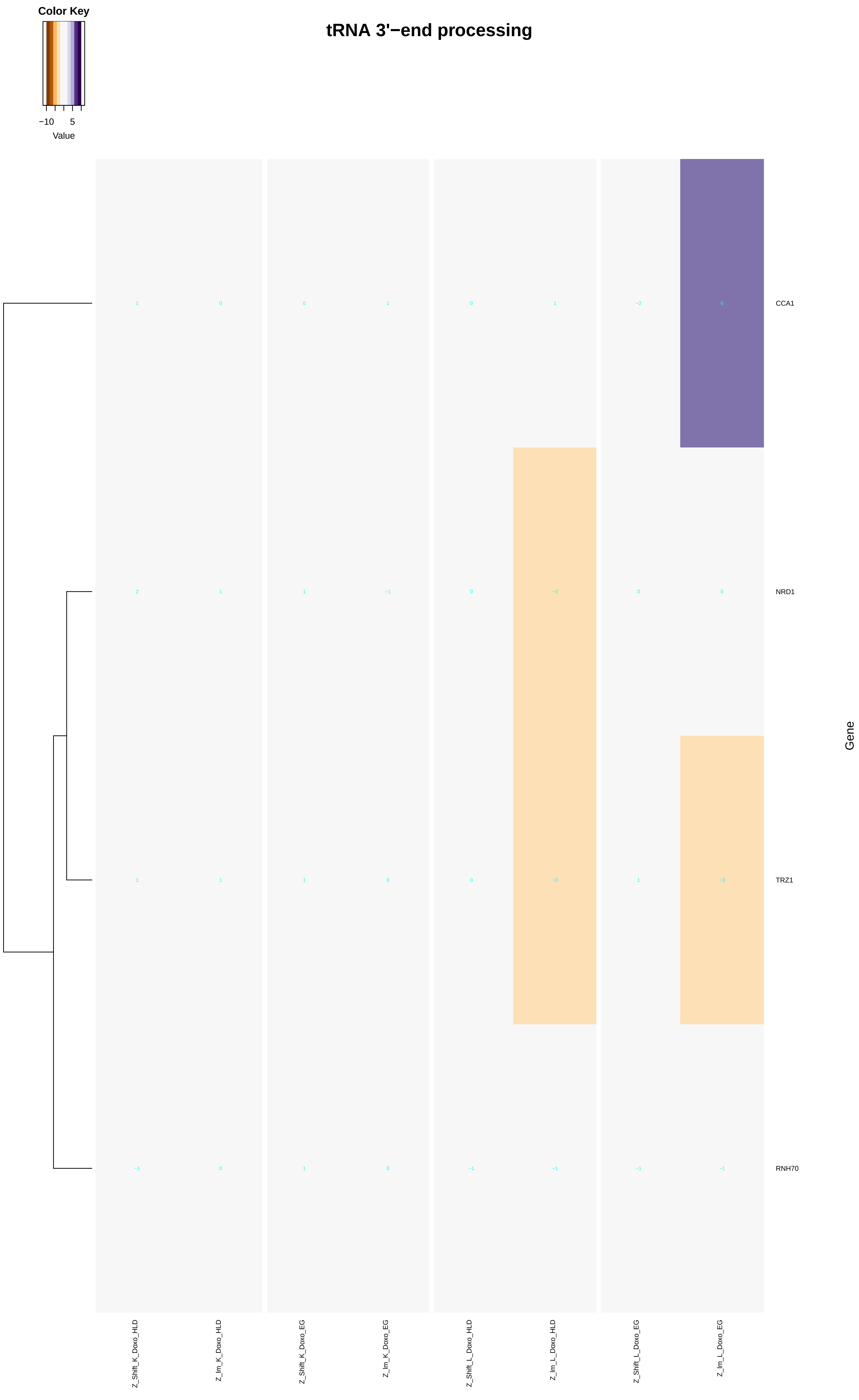
Color Key
−10
5
Value
tRNA 3'−end processing
CCA1
NRD1
TRZ1
RNH70
Gene
Z_Shift_K_Doxo_HLD
Z_lm_K_Doxo_HLD
Z_Shift_K_Doxo_EG
Z_lm_K_Doxo_EG
Z_Shift_L_Doxo_HLD
Z_lm_L_Doxo_HLD
Z_Shift_L_Doxo_EG
Z_lm_L_Doxo_EG

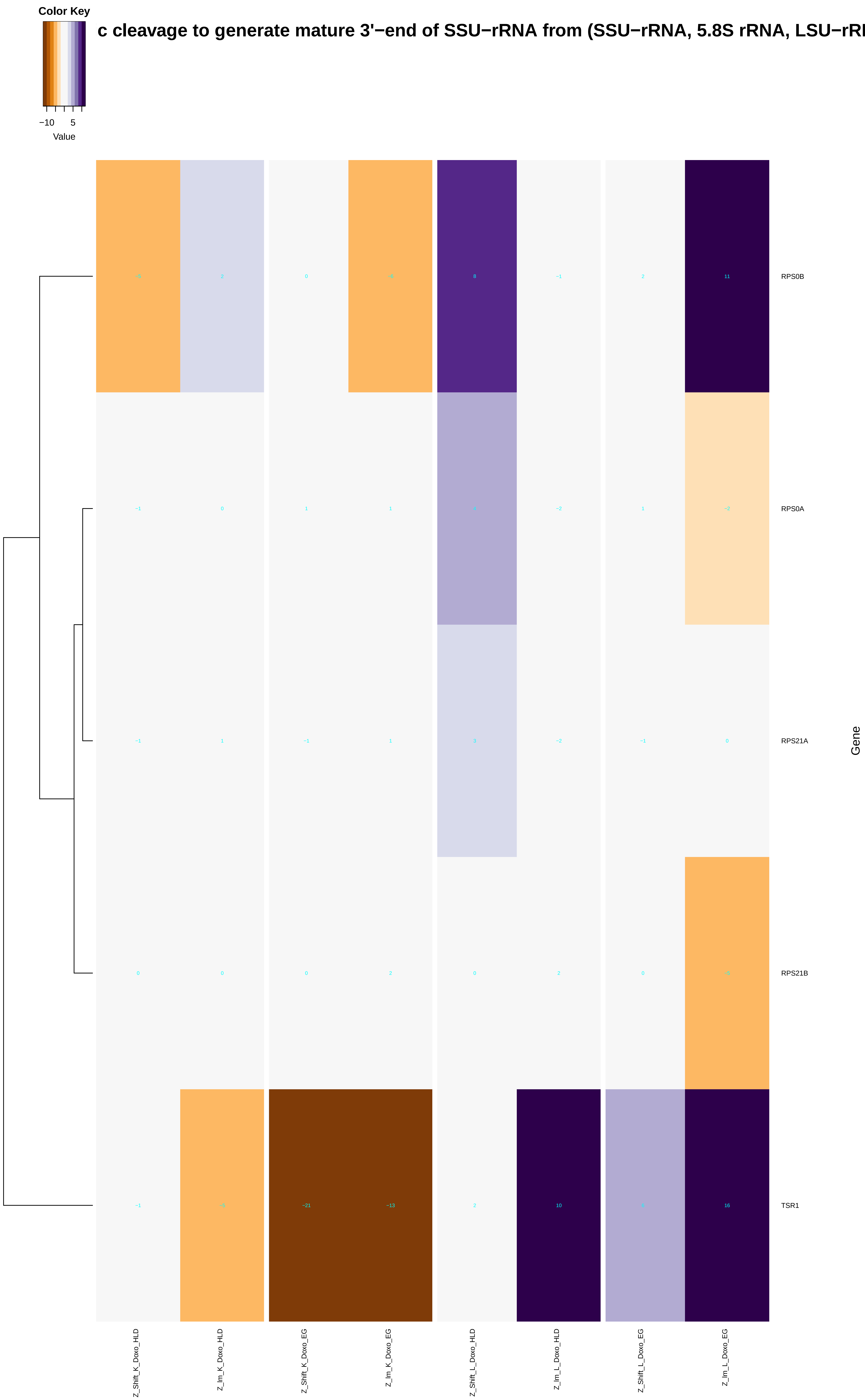
Color Key
−10
5
Value
c cleavage to generate mature 3'−end of SSU−rRNA from (SSU−rRNA, 5.8S rRNA, LSU−rRI
RPS0B
RPS0A
RPS21A
RPS21B
TSR1
Gene
Z_Shift_K_Doxo_HLD
Z_Im_K_Doxo_HLD
Z_Shift_K_Doxo_EG
Z_Im_K_Doxo_EG
Z_Shift_L_Doxo_HLD
Z_Im_L_Doxo_HLD
Z_Shift_L_Doxo_EG
Z_Im_L_Doxo_EG

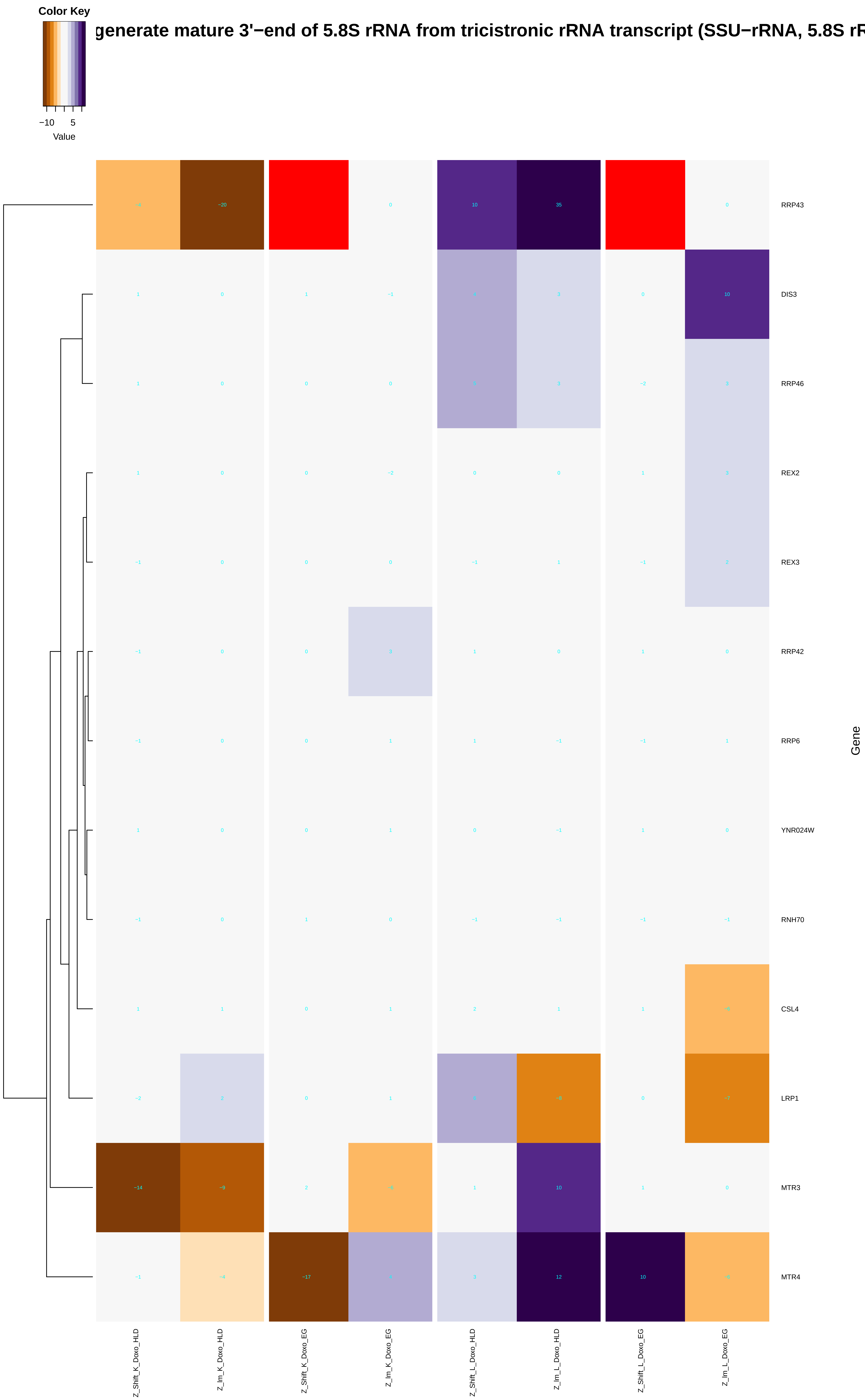

generate mature 3'−end of 5.8S rRNA from tricistronic rRNA transcript (SSU−rRNA, 5.8S rR
Color Key
−10
5
Value
RRP43
DIS3
RRP46
REX2
REX3
RRP42
RRP6
YNR024W
RNH70
CSL4
LRP1
MTR3
MTR4
Gene
Z_Shift_K_Doxo_HLD
Z_lm_K_Doxo_HLD
Z_Shift_K_Doxo_EG
Z_lm_K_Doxo_EG
Z_Shift_L_Doxo_HLD
Z_lm_L_Doxo_HLD
Z_Shift_L_Doxo_EG
Z_lm_L_Doxo_EG

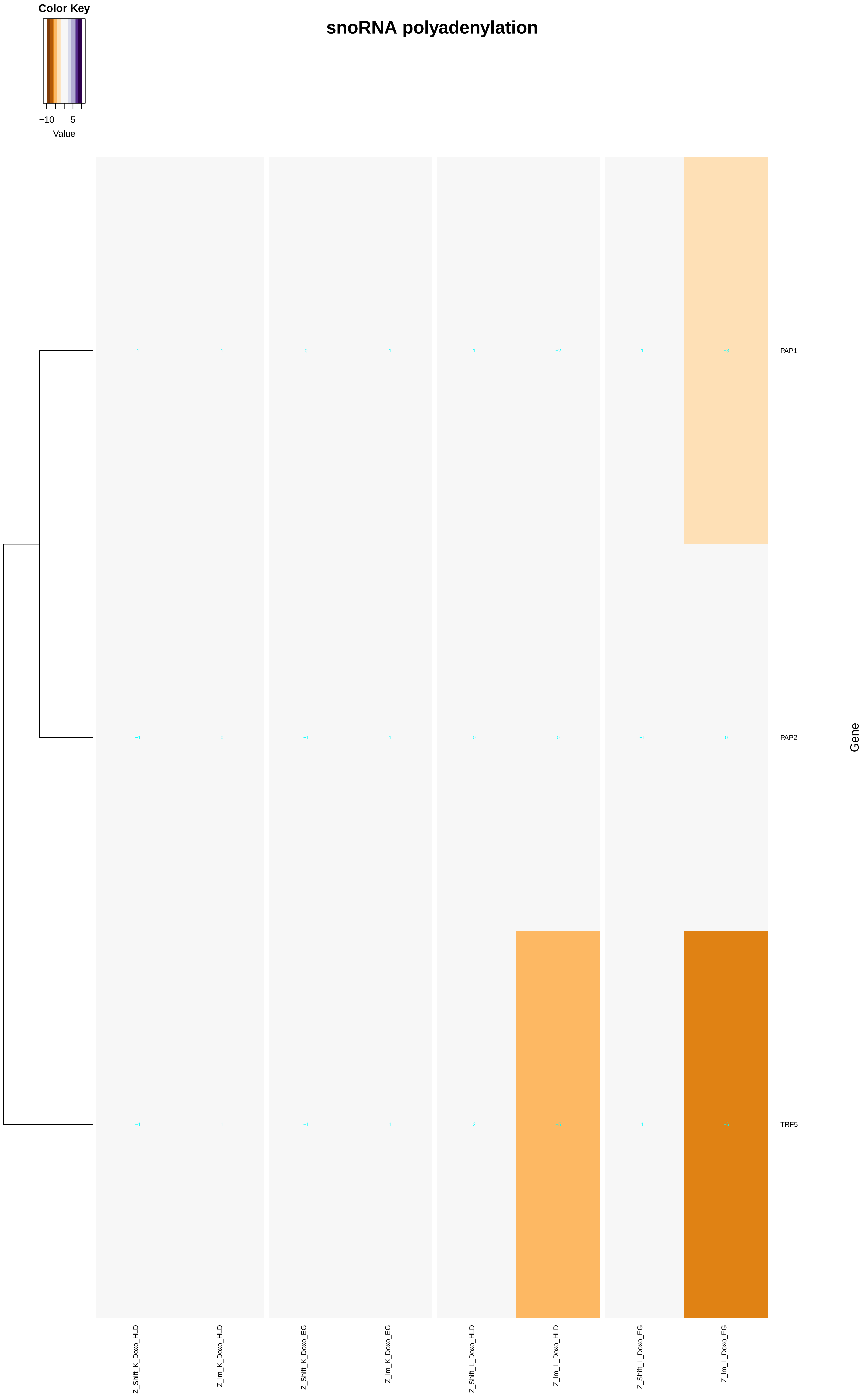

Color Key
−10
5
Value
snoRNA polyadenylation
1
1
0
1
1
−2
1
−3
PAP1
−1
0
−1
1
0
0
−1
0
PAP2
−1
1
−1
1
2
−5
1
−6
TRF5
Gene
Z_Shift_K_Doxo_HLD
Z_Im_K_Doxo_HLD
Z_Shift_K_Doxo_EG
Z_Im_K_Doxo_EG
Z_Shift_L_Doxo_HLD
Z_Im_L_Doxo_HLD
Z_Shift_L_Doxo_EG
Z_Im_L_Doxo_EG

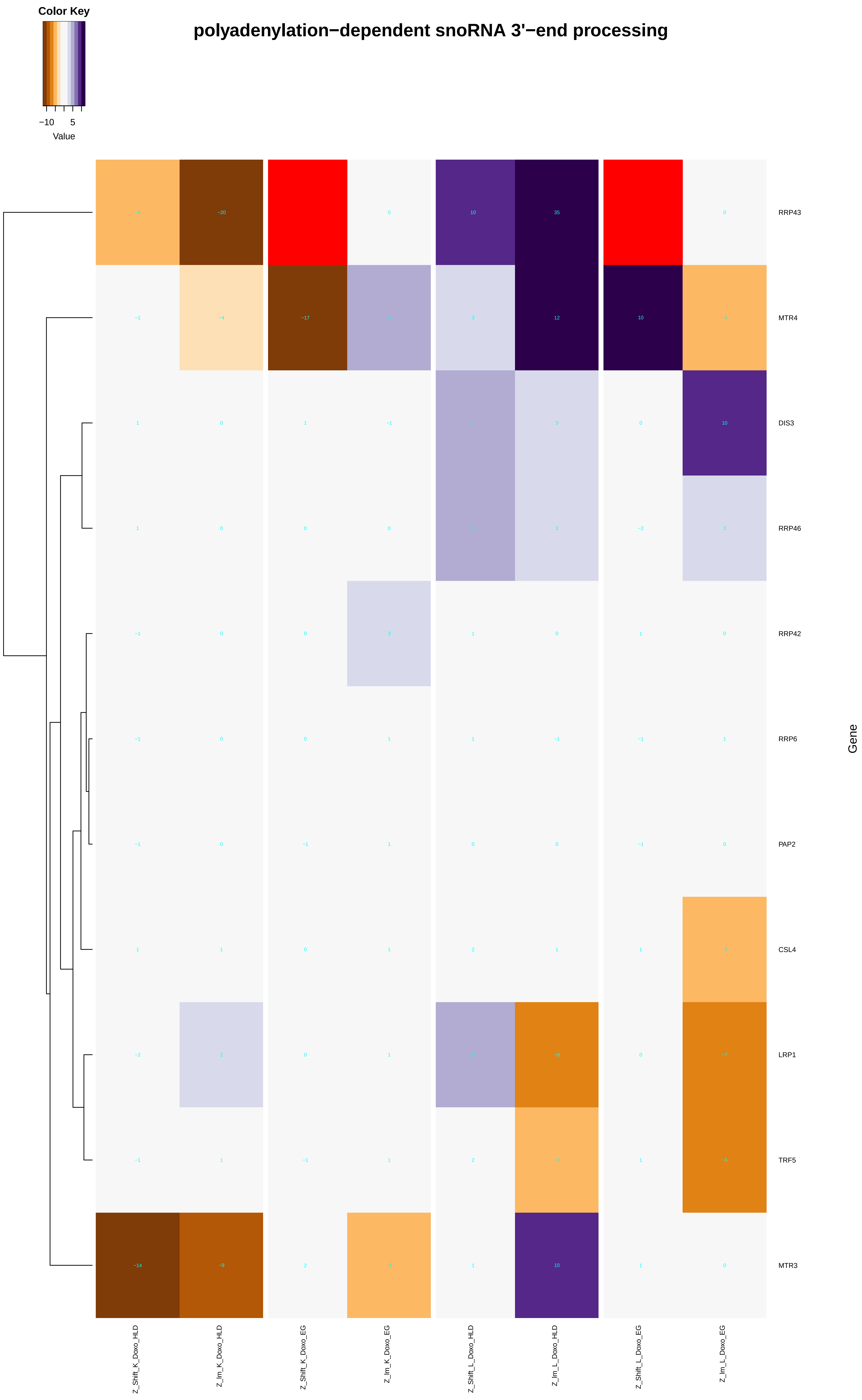
polyadenylation−dependent snoRNA 3'−end processing
Color Key
−10
5
Value
Gene
RRP43
MTR4
DIS3
RRP46
RRP42
RRP6
PAP2
CSL4
LRP1
TRF5
MTR3
Z_Shift_K_Doxo_HLD
Z_lm_K_Doxo_HLD
Z_Shift_K_Doxo_EG
Z_lm_K_Doxo_EG
Z_Shift_L_Doxo_HLD
Z_lm_L_Doxo_HLD
Z_Shift_L_Doxo_EG
Z_lm_L_Doxo_EG

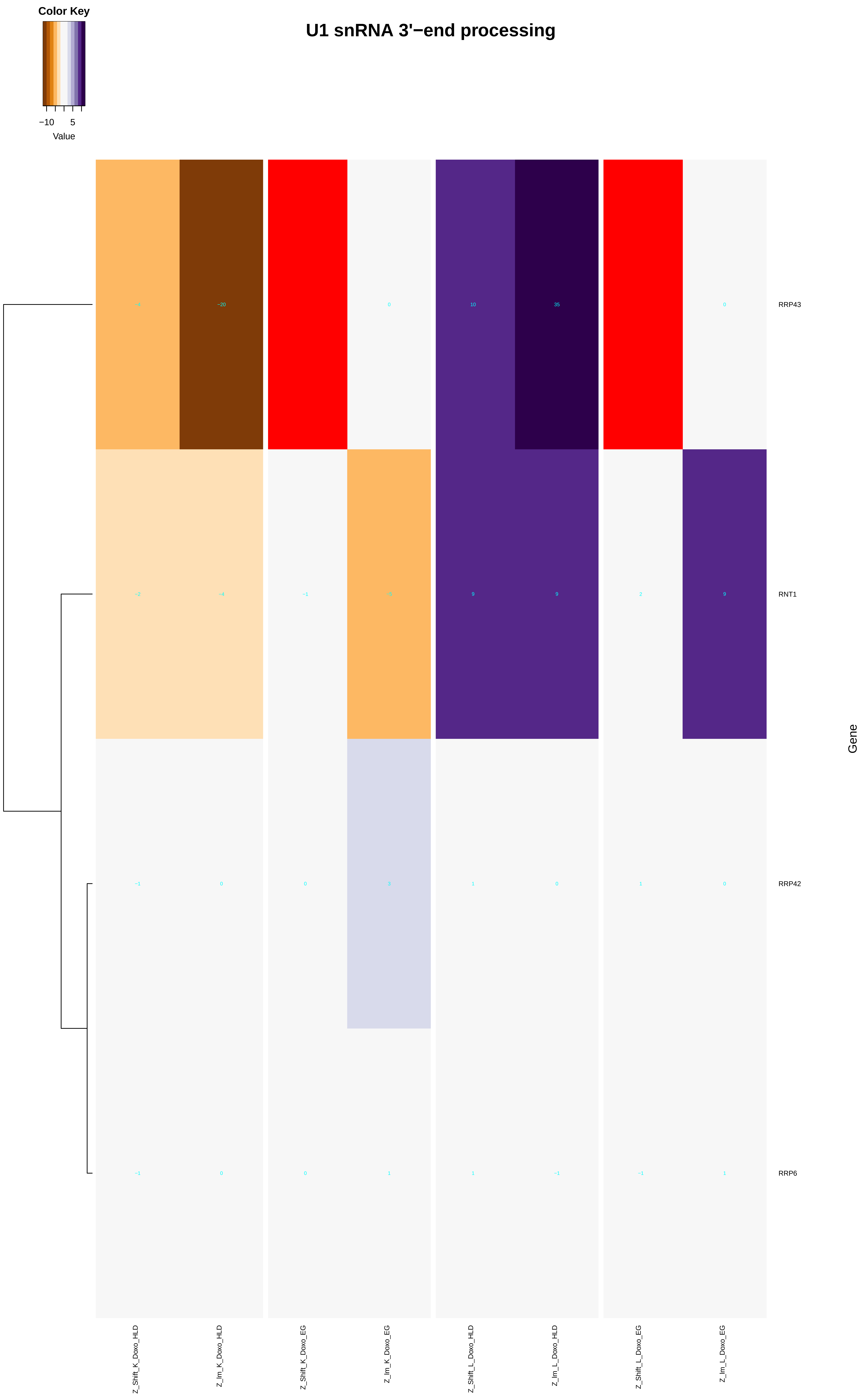
U1 snRNA 3'−end processing
Color Key
−10
5
Value
RRP43
RNT1
RRP42
RRP6
Gene
Z_Shift_K_Doxo_HLD
Z_Im_K_Doxo_HLD
Z_Shift_K_Doxo_EG
Z_Im_K_Doxo_EG
Z_Shift_L_Doxo_HLD
Z_Im_L_Doxo_HLD
Z_Shift_L_Doxo_EG
Z_Im_L_Doxo_EG

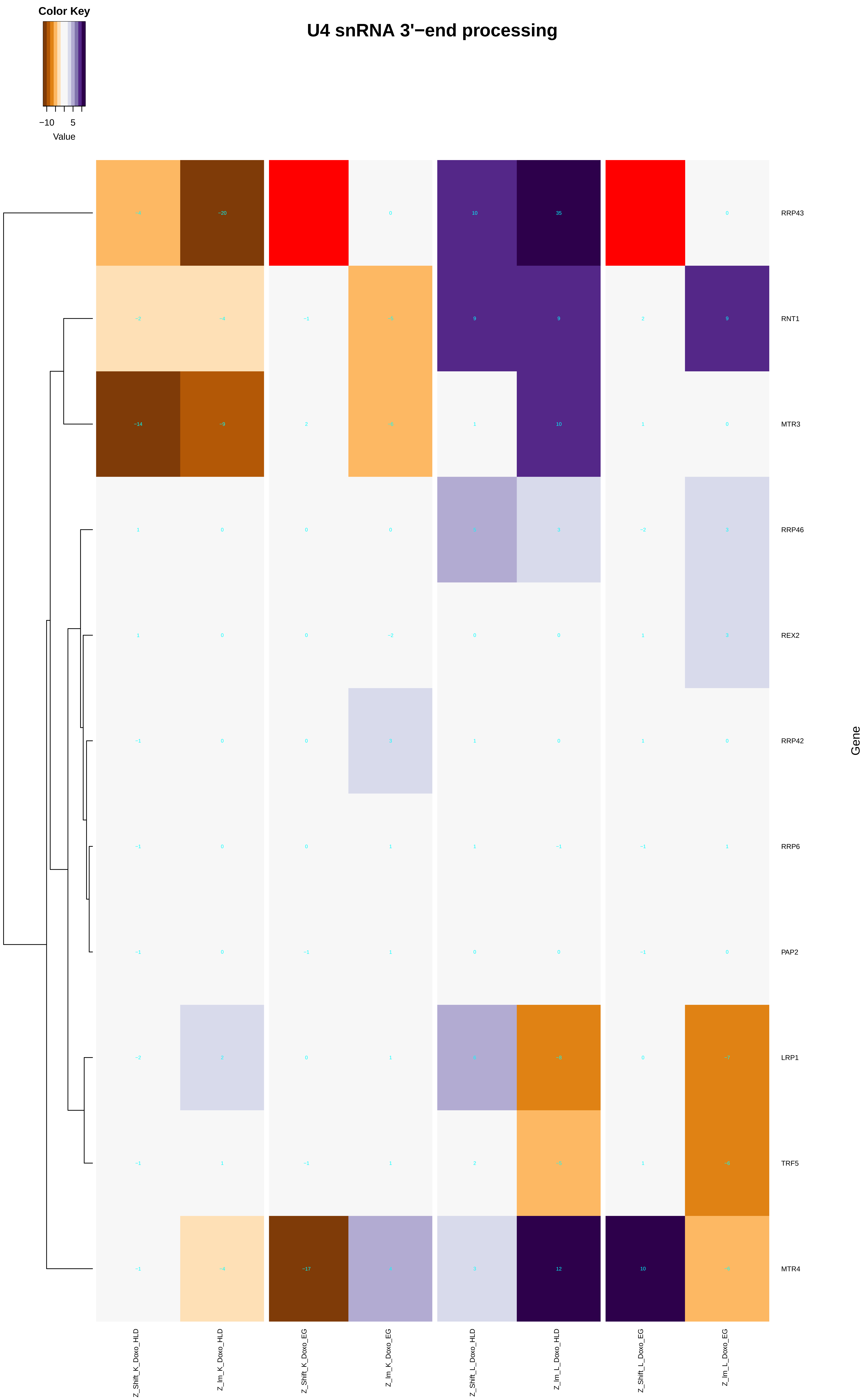
U4 snRNA 3'−end processing
Color Key
−10
5
Value
RRP43
RNT1
MTR3
RRP46
REX2
RRP42
RRP6
PAP2
LRP1
TRF5
MTR4
Gene
Z_Shift_K_Doxo_HLD
Z_lm_K_Doxo_HLD
Z_Shift_K_Doxo_EG
Z_lm_K_Doxo_EG
Z_Shift_L_Doxo_HLD
Z_lm_L_Doxo_HLD
Z_Shift_L_Doxo_EG
Z_lm_L_Doxo_EG

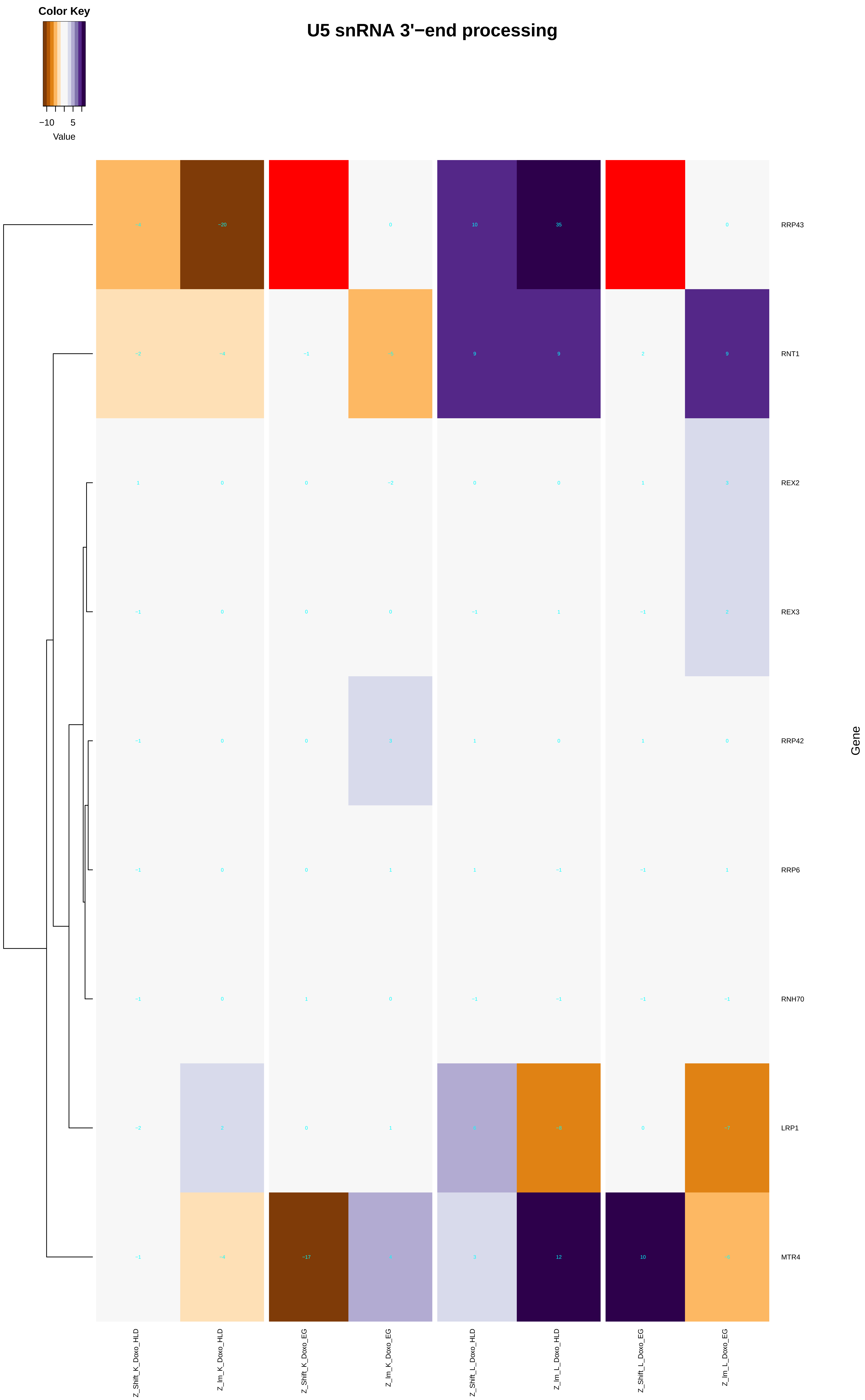

Color Key
−10
5
Value
U5 snRNA 3'−end processing
RRP43
RNT1
REX2
REX3
RRP42
RRP6
RNH70
LRP1
MTR4
Gene
Z_Shift_K_Doxo_HLD
Z_Im_K_Doxo_HLD
Z_Shift_K_Doxo_EG
Z_Im_K_Doxo_EG
Z_Shift_L_Doxo_HLD
Z_Im_L_Doxo_HLD
Z_Shift_L_Doxo_EG
Z_Im_L_Doxo_EG
